# Calm in the midst of cytokine storm: a collaborative approach to the diagnosis and treatment of hemophagocytic lymphohistiocytosis and macrophage activation syndrome

**DOI:** 10.1186/s12969-019-0309-6

**Published:** 2019-02-14

**Authors:** Olha Halyabar, Margaret H. Chang, Michelle L. Schoettler, Marc A. Schwartz, Ezgi H. Baris, Leslie A. Benson, Catherine M. Biggs, Mark Gorman, Leslie Lehmann, Mindy S. Lo, Peter A. Nigrovic, Craig D. Platt, Gregory P. Priebe, Jared Rowe, Robert P. Sundel, Neeraj K. Surana, Katja G. Weinacht, Alison Mann, Jenny Chan Yuen, Patricia Meleedy-Rey, Amy Starmer, Taruna Banerjee, Fatma Dedeoglu, Barbara A. Degar, Melissa M. Hazen, Lauren A. Henderson

**Affiliations:** 1 Division of Immunolgy, Boston Children’s Hospital, Boston, MA USA; 2 Division of Rheumatology, Immunology, and Allergy, Brigham and Women’s Hospital, Boston, MA USA; 3 Division of Hematology-Oncology, Boston Children’s Hospital, Boston, MA USA; 4 Department of Pediatric Oncology, Dana Farber Cancer Institute, Boston, MA USA; 5 Department of Pediatrics, Marmara University Pendik Research and Training Hospital, Istanbul, Turkey; 6 Department of Neurology, Boston Children’s Hospital, Boston, MA USA; 7 Department of Pediatrics, British Columbia Children’s Hospital, University of British Columbia, Vancouver, BC Canada; 8 Division of Critical Care Medicine, Boston Children’s Hospital, Boston, MA USA; 9 Division of Infectious Diseases, Boston Children’s Hospital, Boston, MA USA; 10 Department of Pediatrics, Duke University, Durham, NC USA; 11 Division of Stem Cell Transplantation and Regenerative Medicine, Lucile Packard Children’s Hospital Stanford, Stanford, CA USA; 12 Department of Pediatrics, Boston Children’s Hospital, Boston, MA USA

**Keywords:** Macrophage activation syndrome (MAS), Hemophagocytic lymphohistiocytosis (HLH), Quality improvement research, Evidence-based guideline

## Abstract

**Background:**

Hemophagocytic lymphohistiocytosis (HLH) and macrophage activation syndrome (MAS) were historically thought to be distinct entities, often managed in isolation. In fact, these conditions are closely related. A collaborative approach, which incorporates expertise from subspecialties that previously treated HLH/MAS independently, is needed. We leveraged quality improvement (QI) techniques in the form of an Evidence-Based Guideline (EBG) to build consensus across disciplines on the diagnosis and treatment of HLH/MAS.

**Methods:**

A multidisciplinary work group was convened that met monthly to develop the HLH/MAS EBG. Literature review and expert opinion were used to develop a management strategy for HLH/MAS. The EBG was implemented, and quality metrics were selected to monitor outcomes.

**Results:**

An HLH/MAS clinical team was formed with representatives from subspecialties involved in the care of patients with HLH/MAS. Broad entry criteria for the HLH/MAS EBG were established and included fever and ferritin ≥500 ng/mL. The rheumatology team was identified as the “gate-keeper,” charged with overseeing the diagnostic evaluation recommended in the EBG. First-line medications were recommended based on the acuity of illness and risk of concurrent infection. Quality metrics to be tracked prospectively based on time to initiation of treatment and clinical response were selected.

**Conclusion:**

HLH/MAS are increasingly considered to be a spectrum of related conditions, and joint management across subspecialties could improve patient outcomes. Our experience in creating a multidisciplinary approach to HLH/MAS management can serve as a model for care at other institutions.

**Electronic supplementary material:**

The online version of this article (10.1186/s12969-019-0309-6) contains supplementary material, which is available to authorized users.

## Background

Traditionally, hemophagocytic lymphohistiocytosis (HLH) and macrophage activation syndrome (MAS) were considered distinct conditions managed by hematology/oncology and rheumatology, respectively, with little communication between the two specialties. Increasingly, it is evident that HLH and MAS share an underlying pathophysiology and should be thought of as a spectrum of related conditions. This represents a paradigm shift in our understanding of these disorders and necessitates a multidisciplinary approach to the diagnosis and treatment of patients with HLH/MAS.

HLH may be inherited, as in the case of primary or familial HLH (FHL), or acquired (secondary HLH). All known genetic mutations responsible for FHL reside in genes that code for proteins in the cytolytic pathway, which is employed by CD8^+^ T and NK cells to destroy host cells that are compromised by infection [1]. In the absence of a robust cytolytic response, antigen stimulation persists and drives cytokine production [2–4]. The result is a dramatic presentation in affected patients with fevers, rashes, multi-organ dysfunction, cytopenias, and coagulopathy due to cytokine storm. The diagnosis of HLH is made when a patient is found to have a molecular diagnosis consistent with HLH or fulfills 5 of 8 diagnostic criteria: 1) fever, 2) splenomegaly, 3) cytopenias, 4) elevated triglycerides/decreased fibrinogen, 5) hemophagocytosis, 6) decreased NK cell function, 7) increased ferritin, and 8) increased soluble IL-2 receptor levels [5]. Historically, FHL was diagnosed almost exclusively in infancy and thought to be fatal without chemotherapy and hematopoietic stem cell transplantation (HSCT) [6].

In contrast to FHL, secondary HLH is an acquired condition, typically triggered by a viral infection [7]. Patients with secondary HLH tend to be older and have less severe disease that is often managed with chemotherapy but not necessarily with HSCT [5, 8]. MAS in patients with rheumatic diseases has been increasingly considered a form of secondary HLH [9].

MAS has been described in association with multiple rheumatic diseases; however, it is most frequently observed as a complication of systemic juvenile idiopathic arthritis (sJIA) [9, 10]. MAS is a serious complication of sJIA as it is associated with high mortality rates [9]. As in HLH, MAS is thought to be due to uncontrolled macrophage and T cell activation coupled with exuberant cytokine release, particularly IL-1β, IL-6, IL-18, and interferon gamma (IFNγ) [9, 11–13]. The diagnosis of MAS in the setting of an underlying inflammatory disease can be a challenge. While patients with MAS present with many of the features outlined in the HLH diagnostic guidelines, the HLH criteria do not sufficiently differentiate patients with an active rheumatologic condition from those who have MAS. A recent international effort to create classification criteria for MAS in sJIA has addressed many of these issues and identified fever, thrombocytopenia, transaminitis, hypertriglyceridemia, hypofibrinogenemia, and increased ferritin as important markers of MAS in sJIA. The exact cut-off values for these parameters differ from the HLH diagnostic guidelines because patients with sJIA and chronic inflammation often have elevated inflammatory markers, ferritin, platelet counts, and coagulation studies at baseline [14]. Traditional treatments for MAS have included glucocorticoids, cyclosporine, and intravenous immunoglobulin (IVIG) [15–17]. The advent of biologic therapies that target cytokines has expanded the armamentarium of treatments available for MAS to include medications that block IL-1, IL-6, IL-18, and IFNγ [9, 18–20].

FHL is more closely related to secondary HLH and MAS than previously appreciated. Mutations in FHL genes are found not only in infants with classic HLH features but also in adults with presumed secondary HLH due to infection [21, 22]. Up to 35% of patients with sJIA complicated by MAS have heterozygous variants in FHL genes that are protein damaging [23]. As in HLH, NK cell dysfunction is observed in sJIA patients with MAS and may be due to the deleterious effects of IL-6 [24, 25]. Cytokine excess, particularly IL-18 and IFNγ, is a common feature of both HLH and MAS. IL-18 is dramatically elevated in MAS associated with NLRC4 inflammasomopathies and HLH associated with X-linked inhibitor of apoptosis (XIAP) deficiency [26, 27]. IL-18 is also increased in patients with MAS and HLH without these particular genetic diseases [12, 28, 29]. Interestingly, IL-18 was originally named IFNγ-inducing factor and can promote IFNγ in T cells, which plays a key role in HLH and MAS pathogenesis [30]. In humans with HLH, IFNγ levels are stratospheric, and targeted neutralization of this cytokine improves clinical metrics of the disease [3, 4, 31]. Similarly, high levels of IFNγ and the IFNγ-inducible chemokine CXCL9 differentiate patients with active sJIA from those who have developed MAS [13, 32]. These new findings strongly suggest that HLH and MAS are not discrete diseases but instead represent a continuum of hemophagocytic conditions that share a common pathway of impaired cytotoxicity leading to cytokine storm.

Given the new model of HLH and MAS as inter-related conditions, patients should benefit from a collaborative approach that incorporates expertise from specialties that have traditionally cared for these patients in isolation. We endeavored to utilize quality improvement (QI) methods to facilitate cooperation across disciplines with the goal of developing a diagnostic and therapeutic pathway for patients with HLH/MAS at our hospital.

## Methods

### The EBG model

**Fig. 1:**
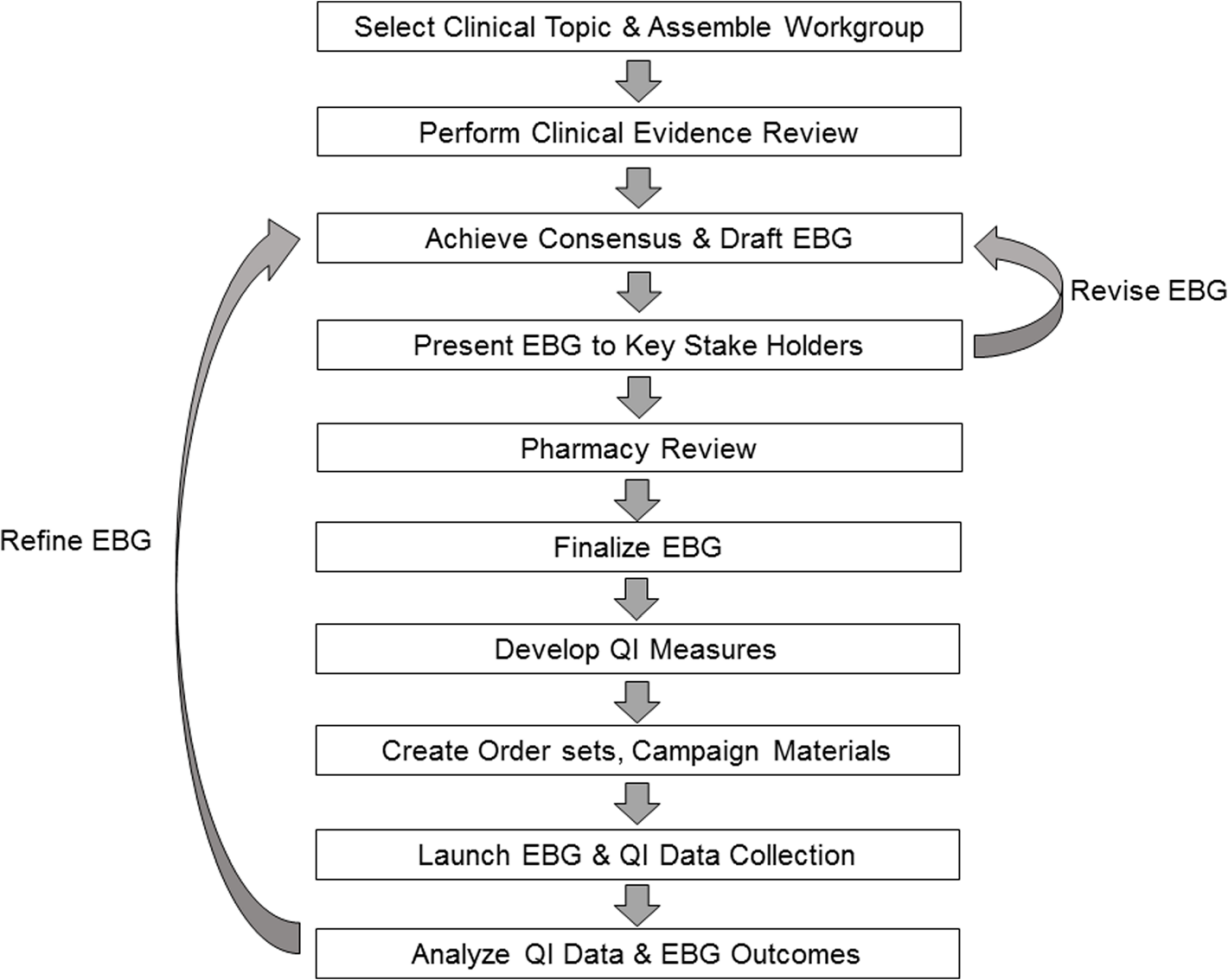
The Evidence-Based Guideline Workflow. The steps required to complete the evidence-based guideline (EBG) process are depicted in the flow chart. QI, quality improvement

To develop a collaborative approach to HLH/MAS management, we enlisted the support of the Evidence-Based Guideline (EBG) program, which is administered through the Department of Pediatrics (DOP) Quality Program at Boston Children’s Hospital (BCH) (Fig. 1). EBGs are clinical algorithms constructed from available evidence in the literature and the expert opinion of locally involved clinicians. The goal is to achieve consensus on how to manage a given condition and thereby reduce practice variability and improve patient care. Data support through the DOP Quality Program allows the outcomes of an EBG to be tracked; therefore, additional iterations of the EBG can be developed for further improvement. The EBG approach has been used to create guidelines for more than 60 clinical topics/conditions at BCH [33].

### Workgroup members

We identified the key subspecialties involved in the management of HLH/MAS and sought a representative from each discipline (Additional file 1: Table S1). Local experts in HLH/MAS were asked to participate by personal invitation. In addition, division chiefs of involved specialties were asked to recommend potential members. Emails were sent to clinicians and trainees in rheumatology, hematology, and oncology specialties particularly involved in HLH/MAS management, to encourage participation.

### Meeting format

HLH/MAS EBG meetings were convened monthly from 3/2016 to 12/2017 (Additional file 2: Table S2). The leads of the workgroup (M.M.H. & L.A.H) served as moderators and identified key questions to address at each meeting. Each session had a pre-set agenda with identified speakers who typically had literature review assignments. At the meetings, the speakers presented a summary of the reviewed literature, which was followed by discussion to build consensus. Based on the nominal group technique, greater than 80% consensus agreement on each point was required in order to pass. At the end of each meeting, areas of agreement and difference were summarized, and action items were identified for the next meeting.

### EBG goals

Prior to the EBG, inpatient consults for HLH/MAS were often channeled through different specialties, most often hematology, oncology, or rheumatology, in a somewhat arbitrary manner. The result was often discrepant diagnostic and treatment recommendations for patients with similar clinical presentations. If multiple specialties were involved in the care of the same patient, clinical recommendations often conflicted, resulting in delayed diagnosis and initiation of treatment. The workgroup sought to eliminate this variability and develop a standard pathway for diagnosis and treatment. The overarching goals of the HLH/MAS EBG workgroup included: 1) Increase communication and collaboration across specialties involved in the care of these patients; 2) Develop an HLH/MAS clinical response team; and 3) Develop a diagnostic and treatment algorithm for HLH/MAS.

## Results

### HLH/MAS clinical response team

In order to implement the EBG, an HLH/MAS clinical response team was created. As a model, the EBG workgroup selected the Kawasaki disease (KD) program, a successful multidisciplinary, clinical team composed of cardiologists and rheumatologists at BCH. As with HLH/MAS, rapid diagnosis and treatment of KD are essential to prevent morbidity and mortality. The EBG workgroup identified key aspects of the KD program that promote success. 1) One subspecialty (rheumatology) is identified as the “gate-keeper” and directs clinical care. 2) Communication is facilitated by an email distribution list. 3) Consensus is achieved before recommendations are given to the house staff. 4) An order set within the electronic medical record (EMR) facilitates diagnostic testing and medication administration.

At BCH, rheumatology has been increasingly engaged in the management of HLH/MAS. Accordingly, the rheumatology consult service was selected to be the “gate-keeper” of the HLH/MAS EBG. Rheumatology serves as the initial point of contact for the house staff to engage when there is concern for HLH/MAS. Rheumatology determines whether the patient should enter the EBG for further diagnostic testing and treatment. An HLH/MAS email distribution list facilitates communication and includes all rheumatology trainees and attendings, as well as representatives from subspecialties involved in the care of HLH/MAS patients (Additional file 1: Table S1). An EMR order set was designed so that diagnostic studies and medications suggested by the HLH/MAS clinical team could be ordered easily by the house staff. Direct links to requisition forms for send-out laboratory studies recommended in the EBG were also included.

### Entry criteria

Entry criteria were identified to help house staff recognize patients with potential HLH/MAS who may be eligible for the EBG. The workgroup reviewed the HLH 2004 diagnostic criteria [5], proposed MAS classification criteria in patients with sJIA [14, 34], and publications on the sensitivity and specificity of elevated ferritin in the diagnosis of HLH/MAS [34–39]. The goal was to capture all inpatients who should undergo a diagnostic evaluation for possible HLH/MAS; hence, entry criteria were designed to be broad (Table 1). Ultimately, fever and ferritin ≥500 ng/mL were selected based on the HLH 2004 diagnostic criteria [5].

**Table 1:** HLH/MAS Evidence-Based Guideline Entry Criteria

Clinical and Laboratory Criteria	
1. Fever (high, unremitting)	
2. Ferritin (≥500 ng/mL)	
3. Other Considerations	
Rheumatologic/hematologic/immunologic conditions that predispose to HLH/MAS^a^	
Neurologic symptoms^b^	
HSM	
Cytopenias^c^	
Hepatobiliary dysfunction^d^	
DIC	
EBV or other viral infection	

*HLH* hemophagocyticlymphohistiocytosis, *MAS* macrophage activation syndrome, *HSM* hepatosplenomegaly, *DIC* disseminated intravascular coagulation, *EBV* Epstein-Barr virus

^a^Including but not limited to systemic juvenile idiopathic arthritis, systemic lupus erythematosus, Kawasaki Disease, familial HLH, lymphoma, Chediak-Higashi Syndrome, Griscelli Syndrome, Hermansky-Pudlak Syndrome type 2, X-linked lymphoproliferative disease 1 & 2

^b^Headaches, cognitive changes, focal examination findings, seizures, findings not explained by degree of illness/medications

^c^Hemoglobin < 9 g/dL, platelets < 200 10^9^/L, absolute neutrophil count < 1000/mm^3^

^d^Elevated liver function tests or bilirubin

At BCH, ferritin is typically obtained as part of the fever of unknown origin evaluation and is often readily available. The workgroup leveraged i2b2, a centralized repository of de-identified clinical data from BCH, to review the number of inpatients within the preceding year with a ferritin ≥500 ng/mL. Twenty-seven patients were identified, a number that was agreed to be reasonably handled by the HLH/MAS EBG.

In addition to fever and ferritin levels, other clinical findings were highlighted to help house staff consider a diagnosis of HLH/MAS: a history of a rheumatologic/hematologic/immunologic disease that predisposes to HLH/MAS, Epstein-Barr virus (EBV) infection, neurologic symptoms, hepatosplenomegaly, coagulopathy, and transaminitis.

### Diagnostic algorithm

**Fig. 2:**
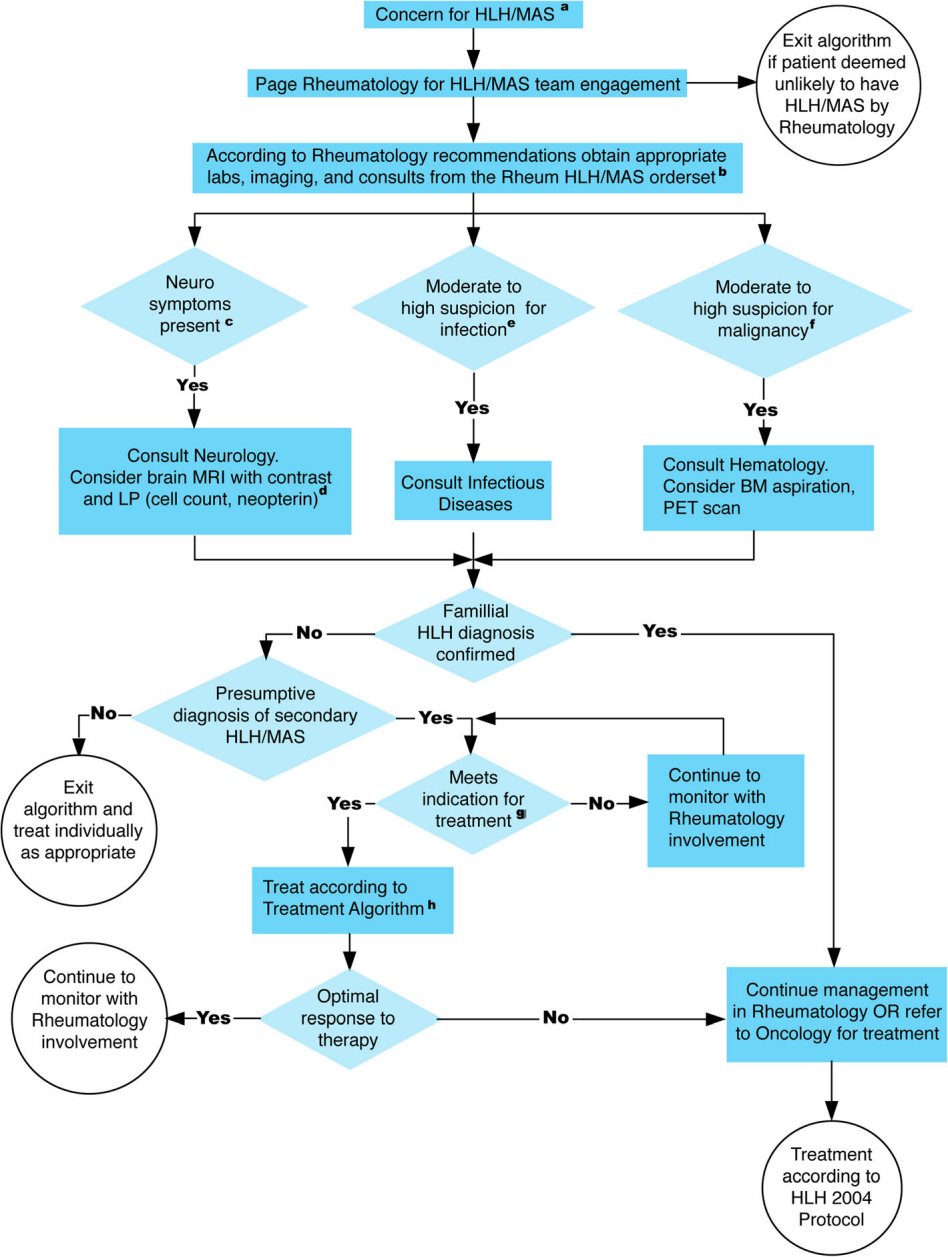
HLH/MAS Evidence-Based Guideline Diagnostic Algorithm. The steps suggested in the HLH/MAS EBG diagnostic evaluation are depicted in the flow chart. HLH, hemophagocytic lymphohistiocytosis; MAS, macrophage activation syndrome; Neuro, neurology; MRI, magnetic resonance imaging; CNS, central nervous system; LP, lumbar puncture; BM, bone marrow; PET, positron emission tomography a. See Table 1. b. See Table 2. c. Neurologic symptoms include headaches, cognitive changes, focal examination findings, seizures, findings not explained by degree of illness/medications.d. MRI findings concerning for HLH/MAS include but are not limited to parenchymal lesions, diffuse brain edema, leptomeningeal enhancement, periventricular white matter changes, brain volume loss, and spinal lesions. A normal MRI does not rule out CNS HLH/MAS. Some patients may only have abnormalities in the cerebral spinal fluid. e. Concern for infection includes but is not limited to immunocompromised hosts, recent travel, known exposures, localizing signs/symptoms, and critically ill patients. f. Concern for malignancy includes atypical lymphadenopathy and cytopenias out of proportion of the clinical presentation. g. Indications for treatment include clinical deterioration, unremitting fevers, progressive worsening of laboratory parameters of HLH/MAS. h. See Table 3 *This guideline was developed for educational purposes only and for use in the Rheumatology Program at Boston Children’s Hospital. Decisions about evaluation and treatment are the responsibility of the treating clinician and should always be tailored to individual clinical circumstances

Once a patient with potential HLH/MAS is identified, the rheumatology team is consulted and determines whether the patient should enter the EBG and undergo a diagnostic evaluation (Fig. 2, Table 2). While the EBG provides recommendations, the diagnostic assessment is at the discretion of the rheumatology consult team.

**Table 2:** HLH/MAS Evidence-Based Guideline Diagnostic Evaluation

Potential Laboratory Evaluation	
CBC w/ diff	
ESR	
Chem 10 (Na, K, Cl, CO2, BUN, Cr, Glucose, Ca, Mg, Phos)	
LFTs (AST, ALT, Tbili, Dbili)	
SPA Panel (IgG, IgM, IgA, C3, C4, CRP, Albumin, Protein)	
LDH	
Triglycerides	
Coagulation Studies (PT, PTT, INR, Fibrinogen, D-dimer)	
Infectious Studies (EBV PCR, CMV PCR, Blood Culture)	
CD107a Mobilization/NK Cell Degranulation	
IL-18	
CXCL9	
Soluble IL-2 Receptor	
Perforin/Granzyme Expression	
SAP/XIAP Expression (Males)	
Genetic Testing for FHL	
Potential Radiologic Evaluation	
Chest X-ray	
Abdominal Ultrasound	

*HLH* hemophagocyticlymphohistiocytosis, *MAS* macrophage activation syndrome, *SPA* serum protein analysis panel, *SAP* SLAM-associated protein, *XIAP* X-linked inhibitor of apoptosis, *FHL* familial HLH

Based on the HLH diagnostic criteria [5] and the ACR/PRINTO 2016 MAS classification criteria [14], laboratory evaluation includes assessment for cytopenias, transaminitis, coagulopathy, and elevated triglycerides (Table 2). Functional and genetic studies commonly used in the diagnosis of FLH are not typically ordered in patients with MAS. Review of the literature by the EBG workgroup demonstrated that mutations in FHL genes and decreased NK cell function are increasingly documented in patients with presumed secondary HLH due to infection and MAS associated with sJIA [21–25]. sJIA patients with MAS are more likely to have variants in FHL-associated genes than sJIA patients without MAS, indicating a potentially more severe disease course that may require more aggressive and sustained therapies [23, 40]. Accordingly, the EBG workgroup agreed that assessment of cytolytic activity with NK cell function and/or CD107a mobilization as well as genetic testing should be considered broadly (Table 2). While genetic testing can take over a month to complete, flow cytometric evaluation of perforin, granzyme, SLAM-associated protein (SAP), and XIAP expression is often available within days and should be considered in patients with high concern for FHL. While bone marrow biopsies are commonly performed in patients with presumed FHL, they are less frequently obtained in children with secondary HLH due to infection or MAS associated with rheumatic disease; therefore, this procedure was not categorically recommended in the EBG.

Cytokine storm is a central feature of HLH/MAS with a particularly important role for IFNγ and IL-18. The EBG workgroup reviewed literature on the importance of these cytokines as supported by data in mouse models and humans [2–4, 12, 26–29, 31, 41–44]. While IFNγ is difficult to measure in the peripheral blood, CXCL9, an IFNγ-inducible cytokine, is easily measured and reflects IFNγ levels. CXCL9 levels are high in HLH and can differentiate patients with active sJIA and MAS [3, 13, 31, 32]. IL-18 is increased in HLH but more so in MAS, where chronic IL-18 elevations are thought to be a risk factor for MAS [12, 28, 29]. Like CXCL9, there is evidence that IL-18 can be used to discriminate between active sJIA and sJIA with MAS [12, 29]. Both CXCL9 and IL-18 are available for clinical testing and are likely biomarkers of HLH/MAS; therefore, these tests were included in the HLH/MAS EBG.

Dedicated EBG workgroup meetings were held with representatives from neuroimmunology (L.B., M.G.) and infectious diseases (G.P., N.S.) to select criteria for involving these subspecialties (Fig. 2). Rarely, children present with HLH/MAS as the first manifestation of underlying malignancy [45]. The EBG workgroup reviewed characteristics of malignancy-associated HLH/MAS in children [45]. In conjunction with input from representatives of oncology (B.A.D.) and hematology (K.W.), criteria for considering additional oncologic evaluation were identified (Fig. 2), reflecting the most common malignancies known to present as HLH/MAS, T and B cell lymphomas.

### Treatment algorithm

Development of a therapeutic pathway for HLH/MAS patients was a major goal for the EBG workgroup. The intent of the treatment algorithm is to provide an agreed upon framework for therapeutic decision-making to reduce practice variability and improve clinical outcomes. Decisions surrounding the start of therapy in HLH/MAS patients are often the most difficult and can be associated with disagreements amongst subspecialists that delay much needed intervention. It is for this reason that the HLH/MAS workgroup focused its recommendations on treatment initiation for the hospitalized patient. The guidelines are meant as suggestions, and clinicians retain their autonomy to deviate from the EBG.

### Treatment algorithm inclusion criteria

The EBG workgroup recommended that FHL patients identified during the diagnostic workup should in most cases receive HLH directed therapy under oncology. In addition, patients with HLH/MAS in the setting of a known malignancy or secondary to chemotherapy were excluded from the treatment algorithm because of insufficient guidance in the pediatric literature and the limited knowledge about safety of biologic medications in this population.

### Treatment initiation

Based on the opinion of the participating clinicians, initiation of treatment should be considered when there is progressive clinical deterioration, worsening laboratory parameters of HLH/MAS, and/or persistent fevers. The workgroup members agreed that treatment should be started as soon as HLH/MAS pathophysiology is recognized and often before the patient meets the full criteria for HLH/MAS. Thus, absolute clinical and laboratory parameters for treatment were not defined. Instead, the trend in the clinical profile is indicative of the need for treatment (Fig. 2), which is in keeping with previously published expert consensus on the dynamics of laboratory values in MAS [46].

### Therapeutic recommendations

Since the EBG recommends treatment at the first sign of HLH/MAS, many patients will not meet diagnostic criteria for HLH or classification criteria for MAS. The use of chemotherapy with agents such as etoposide is not recommended until patients meet diagnostic specifications for HLH. Alternately, medications typically used by rheumatology for MAS are a viable option early in the disease course because they are associated with less toxicity. The EBG workgroup reviewed the literature that demonstrated efficacy for medications such as cyclosporine and anakinra in some patients who meet criteria for not only MAS but also secondary HLH [18, 47–49]. Since these medications have a safer side effect profile than chemotherapy, they were selected as first-line agents (Table 3). An inadequate response to the suggested therapies should immediately trigger re-involvement of oncology to consider more aggressive therapeutic protocols (Fig. 2).

**Table 3:** HLH/MAS Evidence-Based Guideline Treatment Algorithm

Illness Severity	Serious Infection	Potential Medications	Dosing^a^
Moderate	Yes	Anakinra	2–4 mg/kg/dose (max 100 mg) IV/SQ Q12-24 h
		IVIG	1–2 g/kg/dose IV
	No	Anakinra	2–4 mg/kg/dose (max 100 mg) IV/SQ Q12-24 h
		Methylpred	1 mg/kg/dose IV Q12hrs OR 30 mg/kg/dose (max 1 g) IV Q24 hrs × 3 doses
		Cyclosporine (Neoral®)	3–7 mg/kg/day PO Q12hrs
		Tacrolimus	0.1 mg/kg/day PO Q12 hrs
		IVIG	1–2 g/kg/dose IV
Critical	N/A	Anakinra	2–4 mg/kg/dose IV/SQ Q6-24 h (can go higher)
		Methylpred	30 mg/kg/dose (max 1 g) IV Q24 hrs × 3 doses➔1 mg/kg/dose IV Q12hrs
		Cyclosporine	3–7 mg/kg/day PO or 3–5 mg/kg/day IV Q12hrs (enteral preferred)
		Tacrolimus	0.1 mg/kg/day PO or 0.01–0.05 mg/kg/day IV (enteral preferred)
		IVIG	1–2 g/kg/dose IV

*HLH* hemophagocyticlymphohistiocytosis, *MAS* macrophage activation syndrome, *IVIG* intravenous immunoglobulin, *methylpred* methylprednisolone

^a^The medication dosing contained within these guidelines is provided as a reference only. Please refer to institutional formulary or ordering guidelines when placing orders for the clinical care of patients

The workgroup agreed to present a list of recommended medications that could be used alone or in combination for HLH/MAS, depending on the preference of the treating physician. Medication selection and dosing are stratified by the severity of illness and risk of infection, particularly bacterial or fungal infections. The actual assessment of disease severity and infectious risk is made by the treating physician. In moderately ill patients with concern for an underlying infection, less immunosuppressive agents such as IVIG and anakinra are generally preferred. In critically ill patients with uncontrolled cytokine storm (typically patients requiring ICU level care), greater immunosuppression is often warranted despite the increased risk. Drug dose, route, and frequency were based on the pharmacy formulary at BCH and the experience of the involved clinicians.

The EBG workgroup reviewed a number of medications for inclusion in the treatment algorithm. Investigational drugs including NI-0501/anti-IFNγ monoclonal antibody and recombinant human IL-18 binding protein were discussed but ultimately were not included in the EBG because they were not FDA approved at the time of the workgroup meetings and thus were difficult to obtain outside of a clinical trial [31, 44]. Literature on the use of tocilizumab for HLH/MAS was reviewed by the EBG workgroup, including the impact of IL-6 on NK cell function [24, 25], rates of MAS in sJIA patients treated with tocilizumab [9, 50, 51], use of tocilizumab to treat MAS in sJIA and Adult-onset Still’s disease (AOSD) [52, 53], and efficacy of tocilizumab in cytokine release syndrome secondary to T cell engaging oncologic therapies [54, 55]. The reviewed phase III clinical studies demonstrated that MAS can occur in patients with sJIA while on tocilizumab, even with adequate sJIA disease control [9]. In addition, tocilizumab alters markers of MAS/HLH such as C-reactive protein (CRP) and ferritin, making diagnosis difficult [9, 51, 56]. Articles that supported the use of tocilizumab in MAS mostly included patients with sJIA, AOSD, or oncology patients receiving therapies such as blinatumomab and chimeric antigen receptor (CAR) T cells. There was little evidence in the literature for the use of tocilizumab in secondary HLH due to infection. Accordingly, tocilizumab was not recommended as a first-line agent in our treatment algorithm.

Before the era of biologic agents, IVIG, cyclosporine, and glucocorticoids were used regularly to treat rheumatologic-associated MAS [15–17]. Presently, these medications are still used alone or in conjunction with other biologic agents for MAS and have been shown to be effective in HLH [5, 47, 49, 57]. Accordingly, IVIG, cyclosporine, and glucocorticoids were included in the treatment algorithm. Tacrolimus targets the same pathway as cyclosporine and is occasionally prescribed by EBG workgroup clinicians for MAS, particularly in patients who may have difficulty tolerating cyclosporine. As such, tacrolimus was included in the treatment algorithm.

An entire HLH/MAS workgroup meeting was dedicated to discussing anakinra in the treatment of HLH/MAS. Several manuscripts were reviewed by the workgroup that described the successful use of anakinra to treat MAS in sJIA patients, particularly with doses greater than 1-2 mg/kg [9, 18, 19]. In some of these reports, sJIA patients who met the full criteria for secondary HLH still responded to anakinra [18]. In addition, some data supported the efficacy for anakinra in secondary HLH due to infection [48, 58]. Notably, a re-analysis of the original phase III randomized, placebo-controlled study of anakinra for severe sepsis showed that patients with features of MAS (hepatobiliary dysfunction and severe coagulopathy) had improved survival [58]. The HLH/MAS workgroup leaders (L.A.H, M.M.H.) analyzed additional literature in support of anakinra therapy in secondary HLH and MAS [20, 47, 49, 57, 59]. It was unanimously agreed to include anakinra in the treatment algorithm. In the phase III sepsis study, high dose anakinra was used safely; therefore, high dose anakinra was recommended in the treatment algorithm for critically ill patients, even those with suspected infections [58]. There were little data in the literature to support the use of other IL-1-inhibiting drugs in HLH/MAS [9].

### EBG implementation

The EBG was presented to major stakeholders at departmental meetings: rheumatology, immunology, hematology, department of medicine hospitalists, and pediatric interns/residents (Additional file 1: Table S1). The presentations were structured to encourage feedback, which was then incorporated into the EBG. In addition, pharmacy reviewed the suggested medications and dosages to ensure harmonization at the institution level. The created HLH/MAS email distribution list and EMR order set also facilitated implementation of the EBG. In order to increase awareness of the new HLH/MAS EBG, laminated copies were distributed to house staff, email announcements were sent, and the EBG was posted on the DOP intranet site.

### Quality metrics

Since our aim in implementing this EBG was to improve the quality of care provided to this patient population, we defined several quality metrics in concert with the DOP QI team. First, we sought to define our target population and to obtain baseline data regarding these patients prior to EBG implementation. We thus developed a search algorithm for the EMR that allowed us to identify patients who met entry criteria of the EBG and would potentially benefit from its use. The following search criteria were used: ferritin ≥500 ng/mL, fever ≥38.2C, presence of a rheumatology or oncology inpatient clinical note, and clinical note including the term “HLH” or “MAS”. Using these criteria, we evaluated the outcomes of a historical control cohort by pooling the medical records of patients treated within the 2 years prior to EBG implementation. To validate our search strategy, the charts were manually reviewed by rheumatologists who confirmed the presence or absence of HLH/MAS. During the defined time period, a total of 75 patient charts were selected based on the above parameters. After review, 23 patients were confirmed to have the diagnosis of MAS/HLH based on the expert opinion of the reviewing rheumatologist. Prior to EBG implementation, HLH/MAS patients were cared for by different subspecialties: oncology (*n* = 1), rheumatology and oncology (*n* = 2), rheumatology and hematology (*n* = 8), and rheumatology alone (*n* = 10). Treatments for patients diagnosed with HLH/MAS varied and included systemic steroids (*n* = 13), anakinra (*n* = 11), HLH 2004 protocol (*n* = 3), IVIG (*n* = 4), cyclosporine (n = 3), tacrolimus (*n* = 2), infliximab (*n* = 1), and tofacitinib (*n* = 1). These findings underscore the wide range of therapies used for HLH/MAS at our institution historically. The goal of the EBG is reduce this treatment variation and improve outcomes. In order to monitor efficacy of the EBG in achieving these goals, we selected quality measures to be assessed after EBG implementation: length of stay, readmission, time to diagnosis, time to HLH/MAS directed treatment, duration of fever, time to decrease in laboratory parameters of disease activity (ferritin, CRP), need for higher level of care, and death (Table 4).

**Table 4:** HLH/MAS Evidence-Based Guideline Quality Metrics

Quality Improvement Outcome Measures	
Length of hospital stay (days)	
Hospital readmission within 60 days (Y/N)	
Time to HLH/MAS diagnosis (days)	
Time to initiation of HLH/MAS directed therapy (days)	
Fever duration (days)	
Ferritin decrease by 50% of maximum during hospital stay (Y/N)	
Time to decrease of ferritin by 50% of maximum (days)	
Time to CRP < 1 mg/dL (days)	
Need for higher level of care such as ICU/ICP (Y/N)	
Mortality (Y/N)	

*HLH* hemophagocyticlymphohistiocytosis, *MAS* macrophage activation syndrome, *ICU* intensive care unit, *ICP* intermediate care program

## Discussion

Historically, HLH and MAS have been considered distinct diseases that have been separately managed by different subspecialties. As a result of this compartmentalized care, we have observed wide variations in the diagnosis and treatment of HLH/MAS, raising concerns that there may be deleterious delays in recognition and effective treatment of this disease. Over time, however, it has become increasingly clear that these diseases represent a spectrum of related conditions for which an integrated management approach is needed to optimize patient care [9]. Indeed, the need to develop a multidisciplinary approach to MAS has already been recognized. The classification criteria for MAS in sJIA was developed through an international effort that included both pediatric rheumatologists and hematologists [14]. Building on this model of multispecialty consensus, we leveraged QI-based strategies to develop an EBG that presents a unified approach to the diagnosis and treatment of HLH/MAS [33, 60]. Our process provides a model for other institutions interested in improving outcomes for these patients.

Increasingly, there is evidence to support the use of therapies such as IVIG, anakinra, and cyclosporine for patients with secondary HLH and MAS, providing an alternative to chemotherapy-based protocols (HLH 2004 protocol) [18, 47–49]. Kumar et al. proposed a management strategy for adults with secondary HLH that includes first-line treatment with these medications [47]. We extend on this work by developing both a diagnostic and therapeutic algorithm for HLH/MAS in children. A key aspect of our proposal involves collaboration and consensus building with all subspecialties involved in the care of patients with HLH/MAS. We first convened a multidisciplinary work group that included rheumatologists, oncologists, hematologists, neurologists, neuro-immunologists and specialists in intensive care and infectious diseases. Through the EBG workgroup meetings, which included an extensive review of the relevant literature, we developed a consensus approach to patients who raise clinical concerns for HLH/MAS.

Recognizing the complexity of the disease process, we have identified rheumatology as the first-line consultant to help direct the primary team in its evaluation. To streamline our process, we created an EMR-based order set that corresponds to the laboratory evaluation and medications set forth in the EBG. In addition, we created an email distribution list with our HLH/MAS team members, such that real-time multidisciplinary input may be solicited on a case-by-case basis. This distribution list also provides longitudinal contact among team members to foster cohesiveness and inter-specialty collaboration.

Finally, as the EBG represents a QI initiative, we plan to objectively measure its impact on clinical outcomes. Based on a chart review of HLH/MAS patients hospitalized within the 2 years prior to the launch of the EBG, we have identified quality metrics that we seek to improve. Prospective data on these QI measures are currently being gathered. Further, we plan to update the EBG annually to incorporate new developments in the field. For example, laboratory tests such as procalcitonin are increasingly used to differentiate bacterial infections from other sources of inflammation. The HLH/MAS workgroup plans to review infection-related biomarkers for inclusion in the next iteration of the EBG. Future clinical studies may provide insight on the appropriate administration of medications like anakinra in HLH/MAS. In addition, novel therapies are likely to be identified and developed for these conditions. The continuous QI cycle of data gathering, review, and implementation will allow us to modify the EBG to accommodate new scientific information. Finally, we hope to create future versions of the HLH/MAS EBG that address long-term management, tapering of medications, and outpatient follow-up.

We recognize that the specific clinical guidelines outlined in our EBG are intended for use only at BCH. Clinicians at other centers may find that the diagnostic evaluation in our EBG, which includes genetic studies and functional assays, is not feasible for financial or logistic reasons (Table 2). In addition, the proposed treatments may not be available at hospitals outside of North America and Europe (Table 2). We are hopeful that other centers could utilize our approach to develop guidelines appropriate for their institutions. In this way, by providing others with the roadmap to carry out this process, we hope to positively impact the outcomes of patients affected by HLH/MAS more broadly.

## Conclusions

We present a novel approach to the management of HLH/MAS that is built on consensus decision-making across multiple subspecialties involved in treating this patient population. An evidence-based guideline (EBG) for HLH/MAS was created based on literature review and expert opinion that outlines our agreed-upon diagnostic evaluation and first-line treatment recommendations. An implementation strategy was developed to ensure that the recommended HLH/MAS EBG is adopted widely at our institution and outcomes can be monitored. Our approach to developing an HLH/MAS EBG can be used by other clinicians and at other hospitals to build consensus, reduce practice variability, and improve clinical care.

## Additional files

Additional file 1: **Table S1.** HLH/MAS EBG Workgroup Members. (DOCX 14 kb)

Additional file 2: **Table S2.** EBG Workgroup Meetings and Topics. (DOCX 16 kb)

## Abbreviations

AOSD: Adult-onset Still’s disease
BCH: Boston Children’s Hospital
CAR: Chimeric antigen receptor
CRP: C-reactive protein
DOP: Department of Pediatrics
EBG: Evidence-based guideline
EBV: Epstein-Barr virus
EMR: Electronic medical record
FHL: Familial HLH
HLH: Hemophagocytic lymphohistiocytosis
HSCT: Hematopoietic stem cell transplantation
IFNγ: Interferon gamma
IVIG: Intravenous immunoglobulin
KD: Kawasaki disease
MAS: Macrophage activation syndrome
QI: Quality improvement
SAP: SLAM-associated protein
sJIA: Systemic juvenile idiopathic arthritis
XIAP: X-linked inhibitor of apoptosis
