# Supplementary material for: Calm in the midst of cytokine storm: a collaborative approach to the diagnosis and treatment of hemophagocytic lymphohistiocytosis and macrophage activation syndrome
Source: Pediatr Rheumatol Online J. 2019 Feb 14;17:7. doi: 10.1186/s12969-019-0309-6 (PMC6376762; doi:10.1186/s12969-019-0309-6)
Supplement: Supplementary file 1 — Table S1. HLH/MAS EBG Workgroup Members. (DOCX 14 kb) [file 12969_2019_309_MOESM1_ESM.docx]

**Supplemental Tables:**

**Table S1. HLH/MAS EBG Workgroup Members**

| **Sub-Specialty** | **Clinician** | **Fellow/Trainee** |
| --- | --- | --- |
| Oncology | B. Degar, L. Lehmann | M. Schoettler, M. Schwartz, J. Rowe |
| Hematology | K. Weinacht |  |
| Rheumatology | F. Dedeoglu, M, Hazen, L. Henderson, M. Lo, P. Nigrovic, R. Sundel | M. Chang, O. Halyabar |
| ID | N. Surana |  |
| HSCT | L. Lehmann |  |
| Immunology | C. Platt | C. Biggs |
| Neuro-immunology | L. Benson, M. Gorman |  |
| Pediatric ICU | G. Priebe |  |

ID, infectious diseases; HSCT, hematopoietic stem cell transplant; ICU, intensive care unit
