# Supplementary material for: Calm in the midst of cytokine storm: a collaborative approach to the diagnosis and treatment of hemophagocytic lymphohistiocytosis and macrophage activation syndrome
Source: Pediatr Rheumatol Online J. 2019 Feb 14;17:7. doi: 10.1186/s12969-019-0309-6 (PMC6376762; doi:10.1186/s12969-019-0309-6)
Supplement: Supplementary file 2 — Table S2. EBG Workgroup Meetings and Topics. (DOCX 16 kb) [file 12969_2019_309_MOESM2_ESM.docx]

**Table S2. EBG Workgroup Meetings and Topics**

| **Date** | **Topic** | **Outcomes** |
| --- | --- | --- |
| 3/29/16 | Introductory Meeting | Agreed on format of meetings. Major EBG goals identified. |
| 5/10/16 | HLH & MAS Diagnosis | Reviewed literature on HLH diagnostic criteria and MAS classification criteria. |
| 6/14/16 | Ferritin as a Biomarker | Reviewed literature on the sensitivity and specificity of ferritin in HLH/MAS. Reviewed data on number of patients with elevated ferritin levels at BCH. Agreed upon entry criteria for the EBG. LAH and MM assigned to author first draft of diagnostic algorithm. |
| 7/12/16 | HLH/MAS Diagnostic Algorithm | Consensus reached on HLH/MAS diagnostic algorithm. |
| 10/11/16 | Re-Cap Meeting after Summer | Reviewed HLH/MAS diagnostic algorithm. Discussed strategies to develop treatment algorithm. |
| 11/8/16 | HLH/MAS Therapy I | Reviewed literature on anti-IFNγ, anti-IL-18, and anti-IL-6 agents in HLH/MAS. Reviewed medications used and outcomes of sJIA patients with MAS at BHC. Agreed that these drugs should be excluded. |
| 11/17/16 | Immunology Stake Holder Meeting | Discussed preliminary HLH/MAS EBG at the Immunology Clinical Conference for feedback. |
| 12/13/16 | HLH/MAS Therapy II | Reviewed literature anti-IL-1 therapy in HLH/MAS. Data deemed sufficient for use of anakinra. LAH and MM assigned to author first draft of treatment algorithm. |
| 3/31/17 | Rheumatology Stake Holder Meeting | Discussed preliminary HLH/MAS EBG at the Rheumatology Clinical Conference for feedback. |
| 6/8/17 | QI Kickoff Meeting | Introductory meeting with the DOM QI group. |
| 6/30/17 | Review of HLH/MAS EBG with DOM QI Group | Reviewed existing EBG with QI group and agreed the proposal was feasible. |
| 7/25/17 | EBG Implementation Meeting with DOM QI Group | Discussed implementation strategies for the HLH/MAS EBG including order sets, HLH/MAS-dl, and distribution of the EBG document to house staff. Initiated pharmacy review. |
| 9/25/17 | Department of Medicine Stake Holder Meeting | Discussed HLH/MAS EBG with inpatient hospitalists at the Department of Medicine meeting for feedback. |
| 11/3/17 | Hematology Stake Holder Meeting | Discussed preliminary HLH/MAS EBG at the Hematology Clinical Conference meeting for feedback. |
| 11/9/17 | EBG Measurement Meeting with DOM QI Group | Search algorithms developed to identify HLH/MAS patients through the medical records pre and post EBG launch. |
| 11/25/17 | EBG Pre Launch Meeting with DOM QI Group | Finalized HLH/MAS EBG kickoff date and reviewed campaign materials to be used for launch. |
| 12/4/17 | EBG Launch | HLH/MAS EBG Activated |
| 2/5/18 | EBG Metrics Meeting | Reviewed HLH/MAS cases identified by the medical record search algorithm established during 11/9/17. Finalized quality metrics to be used to assess EBG outcomes prospectively. |

EBG, Evidence-Based guideline; FDA, food and drug administration; DOM, department of medicine; QI, quality improvement;
